# Having a real say: findings from first nations community panels on pandemic influenza vaccine distribution

**DOI:** 10.1186/s12889-023-17262-7

**Published:** 2023-11-30

**Authors:** Kristy Crooks, Kylie Taylor, Kiara Burns, Sandy Campbell, Chris Degeling, Jane Williams, Ross Andrews, Peter Massey, Jodie McVernon, Adrian Miller

**Affiliations:** 1 Menzies School of Health Research, Charles Darwin University, Casuarina, NT Australia; 2 Population Health, Hunter New England Local Health District, Wallsend, NSW Australia; 3 Population Health, Hunter New England Local Health District, Tamworth, NSW Australia; 4 Wuchopperen Health Service, Cairns, QLD Australia; 5 Faculty of Health, Charles Darwin University, Casuarina, NT Australia; 6 Australian Centre for Health Engagement, Evidence and Values, University of Wollongong, School of Health and Society, Wollongong, NSW Australia; 7 College of Health and Medicine, Australian National University, Canberra, ACT Australia; 8 College of Medicine and Dentistry, James Cook University, Cairns, QLD Australia; 9 Victorian Infectious Disease Reference Laboratory Epidemiology Unit, The Royal Melbourne Hospital at the Peter Doherty Institute for Infection and Immunity, Melbourne, VIC Australia; 10 Department of Infectious Diseases, The University of Melbourne at the Peter Doherty Institute for Infection and Immunity, Melbourne, VIC Australia; 11 Office of Indigenous Engagement, Central Queensland University, Townsville, QLD Australia

**Keywords:** First Nations, Governance, Pandemic Influenza vaccination, Public health policy, Public deliberation

## Abstract

**Background:**

Recent deliberations by Australian public health researchers and practitioners produced an ethical framework of how decisions should be made to distribute pandemic influenza vaccine. The outcome of the deliberations was that the population should be considered in two categories, Level 1 and Level 2, with Level 1 groups being offered access to the pandemic influenza vaccine before other groups. However, the public health researchers and practitioners recognised the importance of making space for public opinion and sought to understand citizens values and preferences, especially First Nations peoples.

**Methods:**

We conducted First Nations Community Panels in two Australian locations in 2019 to assess First Nations people’s informed views through a deliberative process on pandemic influenza vaccination distribution strategies. Panels were asked to make decisions on priority levels, coverage and vaccine doses.

**Results:**

Two panels were conducted with eighteen First Nations participants from a range of ages who were purposively recruited through local community networks. Panels heard presentations from public health experts, cross-examined expert presenters and deliberated on the issues. Both panels agreed that First Nations peoples be assigned Level 1 priority, be offered pandemic influenza vaccination before other groups, and be offered two doses of vaccine. Reasons for this decision included First Nations people’s lives, culture and families are important; are at-risk of severe health outcomes; and experience barriers and challenges to accessing safe, quality and culturally appropriate healthcare. We found that communication strategies, utilising and upskilling the First Nations health workforce, and targeted vaccination strategies are important elements in pandemic preparedness and response with First Nations peoples.

**Conclusions:**

First Nations Community Panels supported prioritising First Nations peoples for pandemic influenza vaccination distribution and offering greater protection by using a two-dose full course to fewer people if there are initial supply limitations, instead of one dose to more people, during the initial phase of the vaccine roll out. The methodology and findings can help inform efforts in planning for future pandemic vaccination strategies for First Nations peoples in Australia.

## Introduction

The world has now moved beyond the phase of responding to SARS-CoV-2 as a public health emergency of international concern [1]. It is critical to reflect on the appropriateness and impact of pandemic intervention strategies for Aboriginal and Torres Strait Islander peoples (respectfully hereafter First Nations peoples). First Nations peoples are at greater risk of severe health outcomes from pandemics such as influenza and Covid-19 due to culturally unsafe and inequitable healthcare, co-morbidities and lower vaccine uptake [2–6]. Disproportionately increased risk and impacts that are unfair and unacceptable. Governments and health departments need to do better, and this starts with listening [7].

Understanding First Nations people’s views, opinions and preferences in a supportive flexible, respectful and engaging approach can enable First Nations peoples and communities to make challenging and ethically important decisions [8] regarding pandemic vaccinations [9]. Discussions about how to prioritise different groups for scarce resources should take place before a pandemic occurs. In 2018, a group of Australian public health researchers and practitioners developed an ethical framework of how decisions should be made to distribute pandemic influenza vaccine [10], conducted a literature review, modelling [11, 12]; and facilitated citizens’ juries with whole-of-population groups across three Australian locations [13]. This framework described strategies for vaccination goals before and during a pandemic, with three overall aims to support decision-making; (i) creating and maintaining trust, (ii) promoting equity and (iii) focusing on outcomes by reducing harm. The framework emphasised that to achieve these aims there is an obligation to prioritise early vaccine access to some groups by establishing two priority levels (Level 1 and Level 2) for pandemic influenza vaccine allocation. Level 1 prioritises early vaccine access for healthcare workers and First Nations peoples. Decisions about how to prioritise groups within Level 2 would depend on available evidence at the time of, or over time during, a pandemic. Exploring community values to gain deeper understanding of the views and preferences of members of the public was recognised as being important to assess the acceptability of the framework.

To date there is little evidence of engagement with First Nations peoples in pandemic preparedness including around the how pandemic vaccines are distributed when supplies are limited. Different systems and approaches are needed if the values of the ethical framework are to be met as the current health systems in Australia nearly always deliver gaps in health experiences for First Nations peoples [14]. A lack of both engagement and evidence were important factors in the less-than-optimal vaccine rollout of the COVID-19 vaccines in Australia for First Nations peoples [15–17].

The aim for this study was to facilitate two First Nations Community Panels to gain an understanding and provide a description how First Nations communities perceive to be the most appropriate way to distribute limited vaccination resources during the initial phases of an influenza pandemic.

## Methods

The method of enquiry was through a culturally informed design of a citizen jury. Citizens’ juries are used in Australia, [18–20] and globally, [21] to consider in a deliberative way a range of public health policies, however, there has been little work with First Nations peoples specifically using the methodology. Key elements for effective deliberative engagement approaches include providing participants with sufficient factual evidenced-based information; ensuring a range of participant characteristics for diversity of perspectives and opinions; and creating an opportunity for participants to discuss, question and challenge experts on the topic [22]. These factors assume that when people are engaged in high-level conversations about a topic, are provided with factually accurate information and expert opinion, given time to think about the evidence, and speak with experts, people’s views can become more considered and even shift based on the evidence presented and deliberations undertaken. Whilst the citizen jury process is set up like a legal jury, the jury’s decisions are not binding, but can be applied to inform public health policy makers to engage communities to gain an understanding of values, preferences and arguments for and against different approaches and interventions to address a specific problem. Involving people in a high-level conversation about a key public health policy issue can potentially lead to increased public support for the developed policy.

First Nations peoples’ experiences with the judicial system in Australia are often characterised by mistrust, harm and racism [23]. To reduce the potential negative effects of the citizens’ jury method on First Nations participants the jury model was adapted to become a Community Panel. The process of the panels was designed to reflect cultural and community values of the local community where they were held. The adaptation of the process enabled safe spaces to be formed for participants and researchers to be and learn together.

This research was conducted before the COVID-19 pandemic. Although we used a hypothetical scenario to understand First Nations perspectives and preferences on the issue of how to distribute scarce pandemic vaccine, the results could be a way to both evaluate the appropriateness of the COVID-19 response, and to inform suitable planning for future pandemics.

### Study setting

We convened First Nations Community Panels in a large regional town of New South Wales and a regional city in Queensland, Australia. Study locations were chosen because of existing relationships, and work done previously with both locations on other projects. The different locations offered a good contrast. Both Panels were held on two days, 1 or 2 weeks apart, in 2019.

### Panel recruitment

We engaged a local First Nations health care professional in each of the study locations to identify and invite potential participants, through local community and cultural networks. Participants were purposively recruited with consideration of age, gender, family position, household size, community connection and general representation. The Panel process is not structured on representativeness but rather inclusiveness so that participants and their families have a voice at the table. Participants were compensated a total of AUD$400 for their time.

### Community panel process

**Fig. 1:**
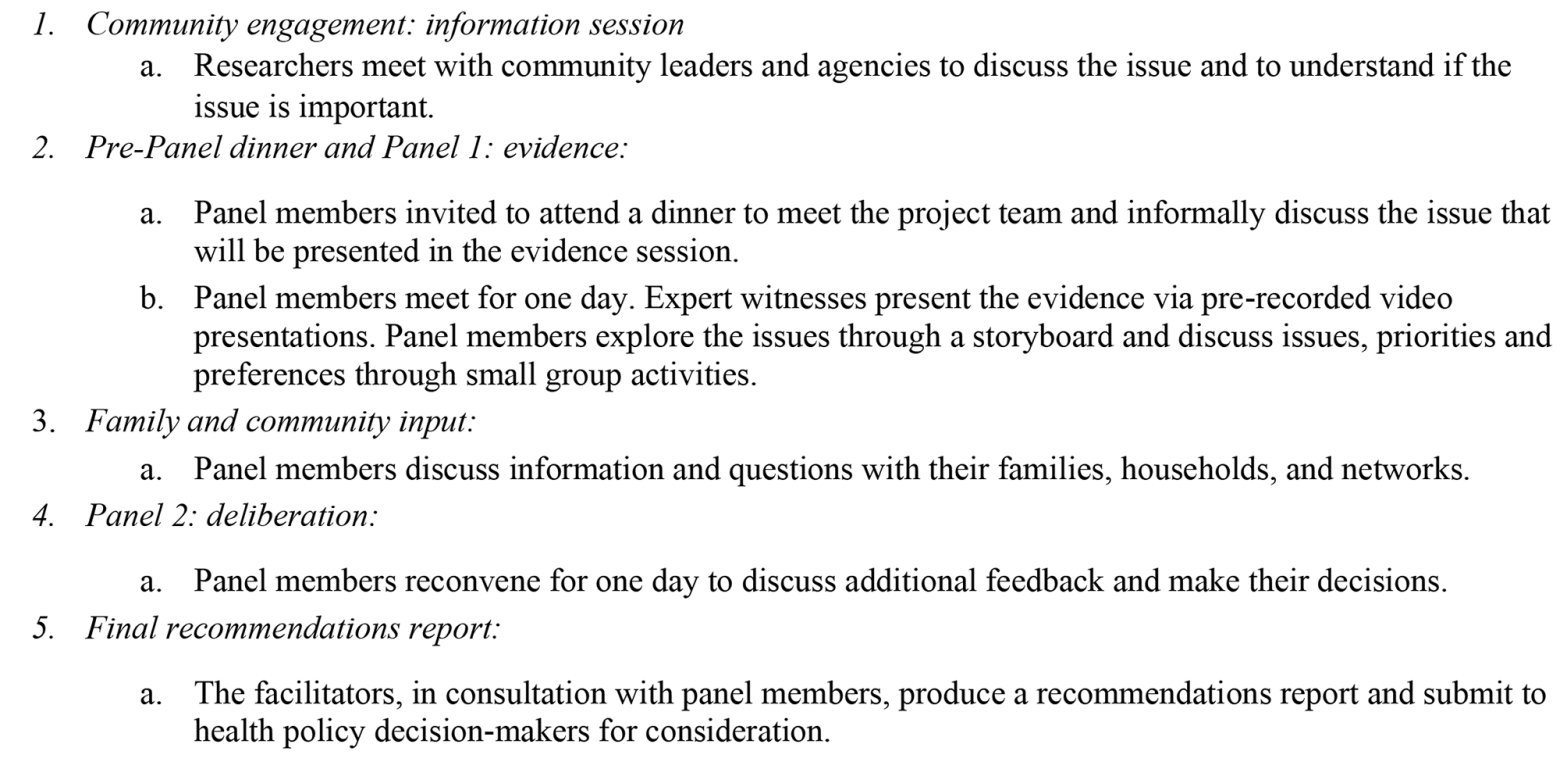
First Nations Community Panel process – pandemic influenza vaccination prioritisation

A five-step decolonising approach to deliberative decision-making was used [24] to understand local contextual issues and to help describe what communities think is the best way to distribute initially limited vaccination resources during a hypothetical influenza pandemic.(Fig. 1).

Both Panels met before the evidence session, to develop rapport and relationships, and to discuss the topic in general terms. During the pre-panel discussions the panel participants talked about their life experiences and challenges faced. Participants were from a range of nations, backgrounds and life experiences and had no direct experience in vaccination strategies.

**Fig. 2:**
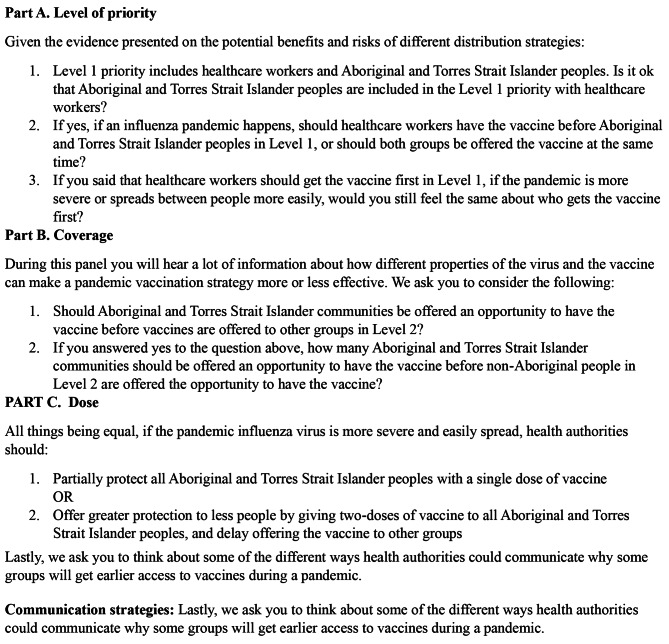
Questions asked of the First Nations Community Panels

Day 1 began with Acknowledgement of Country (showing respect to the traditional custodians on the lands on which the Panels took place), introductions, orientation and expectations of the Panel, the topic and questions,(Fig. 2) and to discuss the process of informed participant consent. Participants received an information booklet outlining the Panel process, and a copy of the expert presentations. On day 1 Panels heard from six First Nations and non- First Nations experts on: (1) infectious diseases and First Nations communities; (2) understanding influenza (the virus, pandemics and the vaccine); (3) the role of vaccines in pandemic response; (4) the impact of vaccines with modelling scenarios; and (5) ethics perspectives on prioritising pandemic vaccines. Experts were selected according to their knowledge, expertise, and professional roles to provide evidenced-based information on the potential problems and benefits of vaccination strategies. Possible strategic examples included providing early vaccine access to First Nations peoples and healthcare workers before other groups; providing more people with partial protection through a single-dose of vaccine; and providing fewer people with greater protection through a two-dose vaccination plan. Expert presentations were shown to the Panels as pre-recorded video presentations. Each presentation was approximately 20-30 min. Following the presentations, the Panels had the opportunity to question the experts (either by telephone or online). A non-First Nations public health expert was present for both Panels to respond to questions and clarification on the issues under discussion. Participants were able to speak with the researchers privately or share comments or questions anonymously or identified on post-it notes during the sessions.

Two First Nations researchers facilitated the Panels. Yarning as a cultural form of conversation is a culturally appropriate way of sharing First Nations worldviews and experiences [25, 26]. Yarning (talking and listening together) was embedded throughout the Panel process for regular check ins with the participants, and allowed time for clarifying the questions, the evidence, or about the topics generally [24]. Divergent views were not a feature as the process of yarning tends towards consensus, but where different views were expressed or felt, participants were given space to sit with these differences, listen some more and work towards a shared understanding. Panel members engaged in small group work, before, during and after the expert presentations to talk about the issues and record their position on each of the questions. A storyboard was used to develop understanding and sharing of key messages and issues, discuss community strengths, concerns, and potential impacts of pandemic influenza. The storyboard and the oral sharing of evidence, stories and understanding helped to reduce written literacy challenges for participants. The health literacy of the participants was high enough to enable participants to question and deliberate with confidence.

At the end of day 1, participants were encouraged to share the information presented and panel questions with their families, households, and social and cultural networks. The Panel discussions then appropriately included the voices of family members not present at the panels. The time between the evidence and deliberation days varied between the Panels, Panel 1 = 15 days in between, and Panel 2 = 6 days. On day 2 of the Panel, the participants reflected on and discussed the evidence with the support of facilitators. The Panel then deliberated together, without the facilitators, to reach a decision for each question.

### Data collection and analysis

Panel discussions were audio-recorded and transcribed, and notes were taken by the researchers. We monitored participants preferences on the questions through anonymous small group activities throughout the Panel sessions. Although the collection points for participant decisions varied for both panels, participants recorded their positions during the Panel; Day 1: (i) morning – before evidence; (ii) afternoon - after evidence; Day 2: (iii) morning – after community input; (iv) afternoon – final decision (verdict). The Panels presented their final decision and recommendations to the researchers. Following the Panel, the researchers in collaboration with the participants, produced a recommendations report outlining the Panels decisions, and justification for their responses.

The findings and report were shared with participants and feedback welcomed. Participants reported being very happy that the final report reflected the position they had reached. Key learnings and concepts from the final report have been presented to policy makers at national conferences and meetings about First Nations pandemic planning.

## Results

We purposively recruited eighteen First Nations participants (QLD, n = 7; NSW, n = 11) through established local, social and cultural networks, with the assistance of a local First Nations Health Worker at the study sites. Participants were from a range of age groups, and most were women (15/18).(Table 1) Women are often the health leaders or champions in families and it is culturally appropriate that this group is given space to be the leaders they are. The Community Panel process enables the voices of families to be heard at the table, so majority female participation does not imply that broader perspectives are excluded.

**Table 1:** First Nations Community Panel member characteristics, 2019

	Community Panel 1 (QLD)	Community Panel 2 (NSW)
**Age (years)**		
18–34	1	1
35–54	2	5
> 55	4	5
**Gender**		
Male	1	2
Female	6	9

*Community Panel decisions*

### Part A: Level of priority

At the end of deliberations, both Panels agreed that First Nations peoples be assigned Level 1 priority pandemic influenza vaccine access. Decisions made were interwoven with social determinants and basic human rights: First Nations peoples should be a priority because of past pandemic experiences and being more at-risk of severe outcomes due to co-morbidities and higher rates of chronic disease. First Nations peoples face barriers to accessing quality and safe healthcare, and infectious disease control strategies do not reflect the social realities of First Nations families, households or communities. Participants stated that *“the way we live, our community structures are against us, we are always there for mob”* and our *“family structures are different, multiple kids in one household, and primary health care approaches must cater to the needs of [First Nations] families and communities.”*

There were differing views over the course of the Panel when asked if healthcare workers should be offered the vaccine first, or if health care workers and First Nations peoples should be offered the vaccine at the same time.(Tables 2 and 3) There was a shift in decisions before and after the evidence and after community input. Panel 1 (QLD) agreed that Healthcare Workers should have the vaccine before First Nations peoples. They reasoned that healthcare workers need to be protected against the disease and are more likely to be exposed to the virus therefore need to be healthy to deliver health and vaccine services.

**Table 2:** Community Panel 1 (QLD) – decisions at different time points during the Community Panel process, October-November, 2019

	PART A – Level of Priority				PART B - Coverage					PART C - Dose	
	Should First Nations people be included in Level 1 priority?		Should Healthcare Workers have the vaccine before First Nations peoples or / Both groups be offered the vaccine at the same time?		Should First Nations communities offered before groups in Level 2?		How many First Nations communities should be offered the vaccine before groups in Level 2?			1 dose	2 dose
	Yes	No	HCW	Both	Yes	No	Some	Half	Most		
#1 morning – before evidence*	6	0	4	2	6	0	2	1	3	3	3
#2 morning – after community engagement & final verdict	7	0	7	0	6	1	0	0	6	0	7

*1 participant was not present for day 1 morning voting

**Table 3:** Community Panel 2 (NSW) – decisions at different time points during the Community Panel process, September-October, 2019

	PART A – Level of Priority				PART B - Coverage					PART C - Dose	
	Should First Nations people be included in Level 1 priority?		Should Healthcare Workers have the vaccine before First Nations peoples or / Both groups be offered the vaccine at the same time?		Should First Nations communities be offered the vaccine before groups in Level 2?		How many First Nations communities should be offered the vaccine before groups in Level 2?			1 dose	2 dose
	Yes	No	HCW	Both	Yes	No	Some	Half	Most		
#1 morning – before evidence	11	0	7	4	10	0	0	2	9	8	3
#1 afternoon* - after evidence	11	0	7	3	10	0	1	1	8	4	6
#2 morning – after community input	11	0	5	6	11	0	0	1	10	3	8
# afternoon – verdict (final)**	10	0	0	11	11	0	0	0	11	0	11

*1 participant left before afternoon voting

** 1 non-vote

Panel 2 (NSW) voted in favour for both healthcare workers and First Nations peoples to be offered the vaccine at the same time. The justification for this decision was to protect those delivering vaccine services which could reduce the spread of disease to the First Nations community; and consideration of protecting those who need it the most such as Elders, children and people who are unable to receive the vaccine. Panel 2 (NSW) had concerns that if First Nations people were offered the vaccine after other population groups that it could widen the health gap between First Nations people and non-First Nations people. There was a level of trust and confidence in vaccine safety among participants. Weighing up different risks were also discussed; a participant summarised this by saying *“which risk are we better to sit with? The risk that we tried, or that we waited?”.* Other issues noted by participants included the risks while waiting for a second batch of vaccines; reducing risk to community by ensuring people administering the vaccines are healthy; and that healthcare workers are aware of universal infection control and may be able to protect themselves more than people in the community. Participants also reflected that First Nations peoples have been used as ‘guinea pigs’ in the past. One participant described the issue of being guinea pigs in these terms *“Do we let the healthcare workers be guinea pigs? We have been guinea pigs our whole lives”.* Participants also reflected on their experience of accessing healthcare, that *“preventative appointments are much harder to get…”*, and more GP education and training about the disease and treatment options like antivirals is needed because *“how many times have we taken a child to the doctor and they tell us it’s a virus, and they send us home. How much awareness is provided to GPs so [people] are not misdiagnosed and missed [an] opportunity to provide some intervention”.*

The Panels offered some recommendations regarding level of priority and distribution strategies:

- Ensure health staff have protective measures in place including personal protective equipment.
- Health staff are provided with antivirals to be able to immunise the community.
- Create a subgroup of Level 1 and prioritize immunisers to have universal protection before all other health staff.

### Part B: Coverage

Both Panels had clear majorities, one by consensus, that First Nations communities should be offered an opportunity to have the vaccine before vaccines are offered to other groups in Level 2. Reasons for this decision centred on the importance of First Nations peoples and culture, and keeping First Nations safe from pandemic influenza through priority vaccination strategies is one way to ensure there is continuation of culture. Additionally, Panels emphasised that First Nations familial structure is diverse, and it is important to protect children, adults and Elders through priority allocation: *“The concept of family is different, is broader, and our worldview is so much different”.* Other reasons for their decision to offer vaccines before other groups were because:

- First Nations people make up a small proportion of the total Australian population, and relative to the number of vaccines offered, should ensure ‘most’ First Nations communities are offered the opportunity to have the vaccine before other groups.
- Values around equity, access and fairness and the need for First Nations peoples to remain a priority population was important. As explained with passion by a number of participants *“for too long [First Nations] people have been trodden on enough”* and *“our people are more vulnerable, low life expectancy, worse outcomes as far as health, therefore [we] should come first”*.
- Key determinants including institutional and individual racism, inadequate housing, barriers to accessing safe and equitable healthcare due to remoteness and lack of healthcare resources negatively impact on First Nations peoples health. Participants also reflected on the impact of the 2009 H1N1 pandemic and the need to prioritize First Nations peoples in pandemic responses.

Despite their decision, Panel 1 (QLD) suggested an overlap of which groups receive the vaccine. The Panel acknowledged that not all First Nations people would want to have the vaccine, and suggested excess vaccines be offered to other high-risk groups in Level 2 at the same time. One participant opposed and maintained their decision for reason being that with all things being fair and equitable *all* people should have the opportunity to be offered the vaccine.

Both Panels supported the strategy that *‘most’* First Nations communities should be offered an opportunity to have the vaccine before non- First Nations people in Level 2 because *“if you can do some, you might as well do all”.* Reasons for this question were similar for both Panels; decisions about vaccine service delivery during a pandemic should be based on geographical location. The Panels recommended that rural and remote communities be prioritized and offered pandemic influenza vaccination services before other areas, because of reduced access to quality and safe healthcare. Continuation of culture and ways of living, being and doing was another reason for this decision; *“our lifestyle, our culture, our health”* need to be protected.

The Panels made additional recommendations regarding offering vaccines to First Nations peoples before other groups in Level 2:

- The term ‘communities’ is broad, ambiguous, and it was recommended that ‘families’ or ‘households’ be used instead. There was acknowledgement that there are non-First Nations peoples living with First Nations families or residing in First Nations communities; and there are non-First Nations households within First Nations communities. There were some ethical dilemmas with this question regarding concerns that non-First Nations people (i.e., partners, parents of First Nations children) living within First Nations families and communities would not be included to receive the vaccine in the initial stages of the vaccination rollout. The Panels, however, maintained their position that First Nations peoples be in Level 1 priority, recognising non-First Nations family/household members would be indirectly protected; *“We are the most vulnerable, we are the most at risk. We are most susceptible to disease.”*
- First Nations adults and children in correctional facilities should be a priority group and be given an opportunity to be vaccinated.

### Part C: Dose strategies

At the end of the proceedings, both Panels unanimously agreed health authorities should offer greater protection to less people by giving two-doses (full course) of vaccine to all First Nations peoples, including peoples incarcerated, and delay offering the vaccine to other groups. Whilst the voting shifted during the course of the Community Panels, two-dose vaccine strategy was preferred for reasons being: two-doses would offer greater protection and maximise immunity; First Nations peoples should be a priority because of greater health disparities for infectious diseases; the First Nations population is small compared to the rest of the population, therefore the vaccine should be offered to all First Nations peoples.

Both Panels raised concerns of the disease being introduced from outside First Nations communities, such as locum healthcare and fly-in-fly-out workers. While remote communities, in their isolation have some protection, a two-dose strategy would offer greater protection. One Panel member reflected on the impact of the 2009 H1N1 on First Nations peoples; *“the first person to die from the swine flu was in remote central Australia… was a [First Nations] person…it’s the lack of quality of healthcare systems that are delivered to remote areas, so we wanted give them the chance of being protected…”.*

Both Panels expressed an ethical dilemma for other ‘vulnerable’, ‘priority’ or ‘high-risk’ population groups missing out; *“Could we give one dose to most, and give two doses to those who are most vulnerable?”* and suggested that antivirals be offered to those eligible to offer protection until non-First Nations people could access the vaccine; *“we haven’t completely forgotten about their wellbeing, want to ensure some level of healthcare delivered to them.”* Participants acknowledged not all First Nations peoples would want to receive the vaccine and recommended that two-doses of vaccine be offered to some of the vulnerable groups in Level 2 such as immunocompromised, elderly, children and pregnant women.

Further recommendations were put forward for this question:

- Encourage individuals to have two-doses of vaccination through incentives.
- Ability for First Nations health workers to administer vaccines during a pandemic.

### Public health communication strategies

The Panels suggested several strategies for health authorities to communicate why some groups would have earlier access to vaccines during a pandemic:

- information around pandemic vaccine prioritisation decisions should come from the government;
- government should share First Nations data and health information to the whole of community to justify why First Nations people are a priority population group in the context of pandemic influenza vaccine prioritisation;
- health education, screening and intervention activities on disease control strategies should be used to promote within the school settings and specific community groups such as mums and bubs groups, and men’s and women’s groups;
- health authorities should engage First Nations networks and Aboriginal Community-Controlled Health Organisations to develop and deliver culturally appropriate messages and information, localized and tailored for First Nations communities;
- information should be disseminated via various forms of mediums including social media, Television, radio, and newspapers;
- deliver pandemic influenza vaccine services in schools;
- equip health staff including general practitioners, nurses, and allied health professionals with education and information to respond to potential community concerns and queries about decisions around vaccine prioritisation.

Other strategies for implementing the vaccine services to First Nations communities were suggested:

- Health authorities use a blueprint from previous successful strategies for health promotion structures to proactively distribute information to First Nations communities.
- Build and strengthen the First Nations health workforce to support a pandemic response through the creation of more positions and broaden the scope of existing roles to deliver health prevention and education programs and activities.
- Utilise existing local community health action and advisory groups, and establish a structure that is community-driven whereby:
  - community identifies issues and strategies,
  - community prioritisation of programs,
  - community implementation, and.
  - communities evaluate the provision of effectiveness of programs.

- Health authorities should target specific groups with a focus on engaging First Nations men.

In addition to the above, further recommendations were put forward for consideration:

- Review and broaden the scope of the First Nations Health Worker role to encompass aspects of public health emergency planning, response and management, that is also inclusive of health promotion and community engagement responsibilities, as well as the ability to be trained in immunisation and administer vaccines.
- Health authorities and local governments develop formal agreements with Aboriginal Community-Controlled Health Organisations to address local needs and formulate a more tailored approach.

## Discussion

### Key findings

Our results show that both Community Panels supported the notion that First Nations peoples should be a priority group in pandemic influenza vaccination allocation, that ‘most’ First Nations communities be offered the vaccination, with a focus on rural and remote communities, [27] and that two-doses of pandemic influenza vaccine be offered to maximise immunity. Both Panels expect the government to distribute public health communication around the reasons for prioritizing some groups over others. Trust was a key value for both Panels and engagement with local First Nations communities is a critical aspect in the development of information, messages and service delivery approaches.

### Vaccination strategies

Vaccination is one way to keep First Nations families and communities protected from severe disease, hospitalisation and death. As health authorities scramble to find ways to address the declining COVID-19 vaccination rates in Australia, particularly among the First Nations population, these Panels provide some guidance on ‘how’ to increase vaccination coverage.

First Nations peoples’ perspectives, voices and solutions should be embedded into pandemic responses, with more targeted approaches to vaccination. Engaging and partnering with First Nations organisations and community views will inform best practice vaccination strategies for First Nations peoples [16, 17, 28–30].

### Vaccine safety

Participants in our study expressed confidence in pandemic influenza vaccine safety. Some studies suggested that First Nations people were less likely to get vaccinated against COVID, [31, 32] have low confidence in vaccine safety and inadequate information about the vaccine [15]. However, while some First Nations peoples had trepidations and expressed concerns about the side effects of COVID-19 vaccine and the time it took to develop the vaccine [16], some were willing to get vaccinated to protect their families and communities [16]. The participants in our study were provided with evidenced-based information and were able to cross-question the experts to gain better understanding of the issue and to make an informed decision. Equipping people with knowledge and accurate health information, in a safe and engaging way, helps the public make informed decisions.

### Health services and first nations health

The establishment of Aboriginal Community-Controlled Health Services (ACCHS) was a positive step toward First Nations self-determination and advancement, creating a safe environment and catering to the needs of First Nations peoples [33]. However, only 40% of First Nations peoples, mostly from remote areas, access an ACCHS [5]. Mainstream health services system and structures are built on colonial foundations, are inherently designed to not be culturally inclusive, are unsafe [4, 34] and often seen as a ‘dying place’, or a ‘last resort’ [34, 35]. There has been a lack of engagement and inclusion of First Nations peoples making decisions about First Nations people’s health, with many strategies being implemented for the whole-of-population that often exclude marginalised, disadvantaged and oppressed populations [35]. Investing in good cultural governance, [36] and building a strong First Nations workforce can make health services a safer place [37], and for services and programs to be developed and implemented based on community needs and priorities [37, 38]. Participants in our study shared experiences of barriers and challenges accessing immunization with families attending a clinic, with only some being eligible to be vaccinated, and others missing out on being vaccinated. Immunization approaches and services need to be consistent, and interpretation and application of immunization policy clear. Primary healthcare approaches to vaccination should be revised, and opportunities to vaccinate the whole family should be offered each time there is an occasion of service at any health facility.

The COVID-19 pandemic has highlighted gaps in on the ground service delivery with some regions upskilling the First Nations workforce to administer vaccines [39, 40]. The Panels strongly recommended a review of the role and scope of the First Nations Health Worker in both State and Community-Controlled health services to encompass aspects of; public health emergency planning and responses; health promotion and education; and to be nationally recognised as trained and qualified immunizers [39, 40]. First Nations health workers play an important role in facilitating trusting relationships and reducing barriers to seeking healthcare and immunizations [41]. Trust and access to culturally safe healthcare are critical for overcoming declining vaccination rates for children and adults [16, 27]. Workforce shortages and reliance on short-term staff has highlighted the need for adequately funded, trained, and accessible workforce particularly in rural and remote communities [27].

### Racism & inequities

COVID-19 has exacerbated institutional racism and health inequities [4, 42] making accessing healthcare more difficult [43]. Health care delivery models changed which meant some people missed out on receiving healthcare. Large vaccination hubs in urban settings provided the public access to a vaccine, while regional and rural areas had to find other ways to distribute the vaccine [29]. Government decisions to redistribute the COVID-19 vaccine allocation away from regional areas to metropolitan areas created anger, mistrust and disengagement in people’s willingness to get the COVID-19 vaccine [44, 45].

### One size does not fit all

Participants in our study recognised the need for more targeted and tailored approaches to vaccination services and programs, and to implement what is already known to work for communities. Pandemic response strategies are often developed for the whole-of-population, which can be problematic for diverse population groups requiring tailored and targeted actions [42]. To enable informed decision making on health policy and practice, First Nations communities must be given reliable and accurate information and time. Gaining a deeper understanding of the contextual issues and potential barriers and enablers for COVID-19 vaccinations can be understood if done within a culturally informed, safe, and appropriate way, that is resourced, flexible and guided by First Nations communities [46].

### Communication strategies

Both Panels provided key recommendations on ways to communicate and build trust with First Nations communities. Specifically, communication strategies and messages and information should come from the government; government should actively engage First Nations health networks and organisations to develop and disseminate key messages in a variety of mediums. Participants in our study accepted that there would be potential backlash and racism about decisions to prioritise some groups over others. The Community Panels were clear in suggesting that government officials and departments should inform the public about vaccine prioritisation decisions, and to educate health staff to then be able to respond to community concerns regarding vaccinations and queries about vaccine prioritisation. Listening to and actioning First Nations peoples’ suggestions and solutions could have informed how the government could have shared information about COVID-19, however, there was a disconnect in the COVID-19 messaging. Public health messaging and information from Government was initially slow during the early phases of the COVID-19 pandemic, and not clear, which caused confusion, distrust, angst and fear [47, 48]. Failing to provide the public with updated, tailored and targeted information caused the public to be wary of what was being communicated and recommended from government officials [45]. Key concerns from the public included; timeliness of the COVID-19 vaccine development; absence of vaccine supply; [45] need for multiple COVID-19 vaccine doses and the language used for the subsequent doses; not being provided with up to date information; and conflicting advice from the Government and from health departments led to confusion about the priority of “opening up” when many priority populations were still under serviced and under vaccinated. This highlights the importance of including First Nations peoples in the decision making about construction and dissemination of pandemic messages and information.

### Engage, listen and act

Although this work explored a way of engaging First Nations peoples in making decisions about pandemic preparedness plans, using a hypothetical scenario, the timing of the Panels coincided with the COVID-19 pandemic response. Including First Nations peoples through decision-making and deliberative approaches, like First Nations Community Panels, can provide government officials and public health policy makers a source of evidence which takes into account First Nations perspectives, views, preferences, decisions and solutions. As the COVID-19 pandemic evolves, it is even more important to understand First Nations peoples’ opinions on COVID-19 vaccinations, antiviral use and long COVID-19. However, there must be a way for public health policymakers to listen and act on the recommendations from First Nations peoples. A formalised mechanism for First Nations voices need to be embedded into public health policy so health authorities and government officials can translate community decisions into practice. Until First Nations peoples are fully involved and engaged in making decisions about pandemic preparedness plans and responses, non-First Nations peoples and leaders will continue to make decisions for, and without First Nations peoples.

#### Limitations

Although our study was small there were many shared experiences, beliefs, values, and perspectives. The community panel and deliberative process enabled First Nations people to have a real and informed say. We acknowledge that the research topic was initiated by public health experts, however the research team engaged with the study sites before undertaking the study to understand the relevance of the topic and sought permission to undertake the research. The informed views of the panels do not represent the views of the many and varied First Nations communities across Australia but the process does elucidate important depth on issues that are potentially life threatening to people.

## Conclusion

First Nations Community Panels demonstrates that when trusted and are provided with evidenced-based information that is delivered in a culturally appropriate and timely way, First Nations peoples can make decisions on complex issues and scrutinise and integrate arguments. Our results show there was shared consensus decision-making, with Panels providing strong and well-reasoned arguments for their decisions. The findings from these Panels provide clear guidance and advice from First Nations peoples on ways to develop engaging, supportive, flexible, and adaptive approaches to pandemic influenza vaccination allocation.

First Nations people want and must continue to be a priority population in all stages of pandemic preparedness and responses. Ongoing and active engagement with First Nations peoples and organisations is necessary to facilitate respectful and trusting relationships to address health inequities. First Nations peoples understand the issues and know what is needed to improve vaccination coverage for their communities and have provided the ‘how’. Systems and structural barriers are root causes to health inequities and disparities. If health authorities and public health policy makers are serious about improving low COVID-19 vaccination rates, government officials must engage, listen, act and respond to community informed solutions and decisions. Although this work explored a hypothetical scenario of prioritization of pandemic influenza vaccination allocation, the results, particularly relating to communication strategies, utilising and upskilling the First Nations health workforce, and targeted vaccination strategies, also have direct relevance for culturally informed and appropriate COVID-19 vaccination distribution strategies for First Nations communities in Australia.

## Data Availability

Data are available on reasonable request. Data sharing protocols are underpinned by Indigenous Data Sovereignty principles, in line with agreed community protocol and distribution of the data. Data include materials from Community Panels. Therefore, data may be available under reasonable request and will require Community Panel permission. Contact person Kristy Crooks: Kristy.crooks@students.cdu.edu.au.
